# Transformation of a Ferry Ship into a Ship Hospital for COVID-19 Patients

**DOI:** 10.3390/ijerph17238976

**Published:** 2020-12-02

**Authors:** Paolo Cremonesi, Marina Sartini, Anna Maria Spagnolo, Giulia Adriano, Eva Zsirai, Carlotta Patrone, Isabella Cevasco, Maria Luisa Cristina

**Affiliations:** 1 Department of Emergency Medicine, Galliera Hospital, Mura delle Cappuccine 14, 16128 Genoa, Italy; paolo.cremonesi@galliera.it; 2 S.S.D. U.O. Hospital Hygiene, Galliera Hospital, Mura delle Cappuccine 14, 16128 Genoa, Italy; anna.maria.spagnolo@galliera.it (A.M.S.); maria.luisa.cristina@galliera.it (M.L.C.); 3 Department of Health Sciences, University of Genoa, Via A. Pastore 1, 16132 Genoa, Italy; 4 Hospital Infection Control Committee, Galliera Hospital, Mura delle Cappuccine 14, 16128 Genoa, Italy; giulia.adriano@galliera.it; 5 Galliera Hospital, Mura delle Cappuccine 14, 16128 Genoa, Italy; kormy@hotmail.com; 6 Office Innovation, Development and Lean Application, Galliera Hospital, Mura delle Cappuccine 14, 16128 Genoa, Italy; carlotta.patrone@galliera.it; 7 Healthcare Professions Structure, Galliera Hospital, Mura delle Cappuccine 14, 16128 Genoa, Italy; isabella.cevasco@galliera.it

**Keywords:** COVID-19, ship hospital, public health and environmental policies

## Abstract

Liguria is a northwestern region of Italy that, since the WHO has declared COVID-19 as a pandemic (11 March 2020), presented 108 patients hospitalized, 34 of which were in the intensive care unit. Due to this serious epidemiological emergency, the transformation of a long-distance ferry ship into a hospital ship for COVID-19 patients who were still positive after the acute phase of the illness was carried out to free up hospital beds for patients in the acute phase. The ship was moored in the port of Genoa, the capital of Liguria. The conversion was localized to a single deck, where designated healthcare areas were identified. From 23 March to 18 June 2020, 191 patients were admitted onto the ship; they were provided with high-level healthcare guaranteed by the multi-disciplinary nature of clinical competencies available. Patients had a favorable outcome in all cases, confirmed by their recovery and negative swab results. Moreover, no cases of voluntary discharge were recorded. To the best of our knowledge, this is the only example in the world in which a passenger ship was transformed into a ship hospital for COVID patients.

## 1. Introduction

Coronavirus disease 2019 (COVID-19), caused by severe acute respiratory syndrome-coronavirus 2 (SARS-CoV-2), has become a major health problem causing severe acute respiratory illness in humans [1]. Since its first identification in Wuhan, China, in December 2019 [2,3,4,5,6], it has spread rapidly worldwide [7,8,9], although the Chinese government implemented a number of severe restrictions on people’s movement in an attempt to contain its local and international spread [10].

On 11 March 2020, the WHO characterized COVID-19 as a pandemic [11]. Italy has had 12,462 confirmed cases, according to the Italian National Institute of Health, as of 11 March, and 827 deaths [12,13]. The percentage of patients in intensive care reported daily in Italy between 1 March and 11 March 2020 has consistently been between 9% and 11% of patients who are actively infected. The number of patients infected since 21 February 2020 in Italy closely follows an exponential trend. Forecasts for that month estimated that if the trend continued, within a week, there would have been 30,000 infected patients, intensive care units would have reached bed place saturation, and up to 4000 hospital beds would be needed by mid-April 2020 [13]. At a national level, the epidemic explosion affected Northern Italy the most, right from the outset. Liguria is a northwestern region of Italy that, since the WHO declared a pandemic, presented 185 positive cases, with 108 patients hospitalized, 34 of which were in intensive care. On 31 March 2020, a total of 1443 bed spaces were available in the Liguria Region (Data source: Ligurian Healthcare Agency, A.Li.Sa.). Due to the serious epidemiological emergency and forecasts of a possible explosion in the number of cases, at a regional level in Liguria and in other seriously affected regions, there was an urgent need to create bed spaces for intensive care patients. Therefore, infected patients requiring lower intensity care, discharged from hospitals in the Liguria Region and not yet able to return home, needed to be directed to alternative facilities.

This paper describes the transformation of a long-distance ferry boat (G.N.V. Company) into an isolation and care facility for COVID-19 patients who were still positive after emerging from the acute phase of the illness, with the purpose of sharing some of the care burden of local hospitals and to free up bed spaces for patients in the acute phase of the disease. The implementation of this project and running of the hospital ship was initially made possible thanks to the cooperation of local hospitals in the metropolitan area of Genoa, successively with the contribution of healthcare authorities from the rest of the region. The article is structured in two different sections, one of which describes the main transformations of the ship into a hospital ship, and the other the infection risk management procedures adopted.

## 2. Description of the Selected Ship and Its Transformation into a Hospital Ship

**Figure 1:**
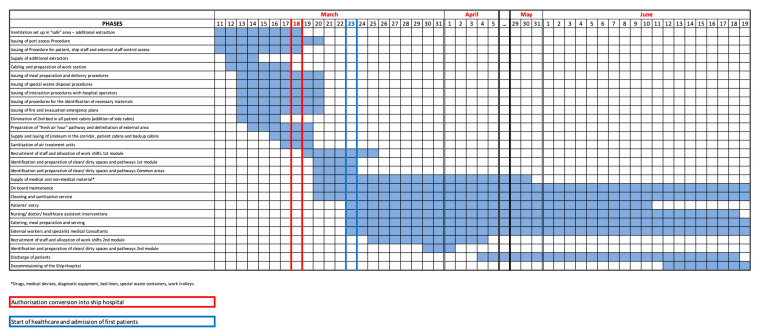
Time frames of major preliminary actions undertaken to turn the ship into a hospital ship and actions undertaken to enable the provision of healthcare.

The ship identified for this purpose was moored in the port of Genoa, a port city with 841,180 inhabitants in its metropolitan area [14], and is the capital of the Liguria region. The ship was fitted with 567 guest cabins in total, of which there are two triple cabins for the infirmary and one isolation cabin with two bed spaces. The conversion of the long-distance ferry boat into a hospital was completed in a very short space of time. The region of Liguria granted authorization on 18 March 2020, and five days later the first module of cabins for COVID-19 positive patients was ready, guaranteeing facilities for admissions. Figure 1 illustrates a time line of the main actions for the transformation of the ship into a hospital ship and actions executed to enable the provision of healthcare up to the moment of the hospital ship’s gradual decommissioning.

### 2.1. Air Handling and Ventilation

Regarding the ventilation system, the ship was already fitted with 30 air treatment units (ATUs) (air conditioning and extraction), eight of which were dedicated to cabin groups. The ship was divided into main vertical zones, which were separated in terms of both structural and ventilation systems. Public areas and staircases were fitted with air treatment systems that did not communicate with the cabin ones. Kitchens for crew members and passengers and the on-board hospital were all fitted with independent ATUs. Each passenger cabin was fitted with an air inlet through a conditioner that supplied air from the outside and/or the recirculation grills installed in corridors. Each was also fitted with an extraction system, from the cabin rest room to outside the ship, directly to the chimney stack area and far from air aspiration equipment to avoid short circuits between safe and unsafe zones. The added extractors have created a negative pressure inside the patient cabinets, providing an additional safety factor.

### 2.2. Patient Selection, Pre-Triage, Admission, and Discharge

SARS-CoV-2 positive patients had to satisfy certain requirements in order to be eligible for admission, which the discharging acute care ward was required to guarantee by filling out a specific pre-admission assessment sheet (Table 1). Patients suffering from serious psychiatric disorders, a total lack of self-sufficiency, or senile dementia were not eligible for admission on board.

Patients were selected at the ship on the basis of their possession of characteristics listed as eligibility criteria in the assessment document. The medical scientific head, tasked with the coordination of local discharging hospitals and the hospital ship, selected patients and coordinated their transport with the Genoa 118 Ambulance Service, notifying admitting doctors of their arrival. Upon ambulance arrival and on the basis of received information, hospital ship nursing staff ran a further check of the admission candidate’s characteristics in a protected area of the dock prior to admission on board along “flow 1”. This occasion was also used to provide the patient with information on conduct and behavior requirements. Prior to notification of Deck 7, the recruited patient was accompanied to the cabin by a nurse and healthcare assistant, who sanitized the pathway in the transiting patient’s wake to ensure the full safety of the next patient and accompanying medical staff, as well as external providers. After checking the patient in, crew staff would notify the entire ship of the patient’s transit by intercom so as to free pathways and safeguard staff. Once the patient entered the ship, healthcare workers, each within the scope of their own competencies, managed patient admission. During their stay at the ship hospital, patients received blood chemistry test analysis, clinical assessment, and treatment. Healthcare activities were provided in a synchronous way and organized according to pre-established sequential procedures between the safe and unsafe zones, reducing the risk of contamination. Patient discharge was subject to a negative swab result, in accordance with provisions set forth in regional healthcare directives. A healthcare assistant cooperated with other operators for discharge procedures and followed the patient along the checkout pathway (up to the ship’s exit), activating protected discharge pathways if necessary.

### 2.3. Access for Healthcare Staff/Patients/Providers

**Figure 2:**
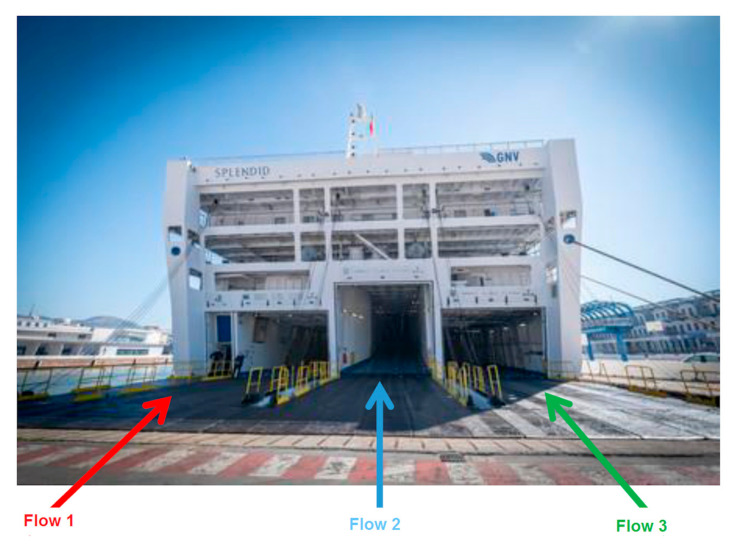
Entrance for healthcare and non-healthcare staff, patients, and providers.

Three different pathways were identified (Figure 2) for:

- Healthcare staff, patients/inpatients, firms, and healthcare service providers (left side stern gangway—Flow 1).
- Ship and pantry supplies (Central gangway—Flow 2).
- Crew/GNV staff and external staff, for occasional technical interventions (right side stern gangway—Flow 3).

Flow 1 was further divided into two areas/entrances:

- Left side (water side): main entrance for hospital ship healthcare staff and clean material.
- Right side (adjacent to Flow 2): patient transport by ambulance and dirty material.

### 2.4. The Designation of Areas on the Ship Hospital

The conversion of the ferry ship into a COVID hospital-ship was localized to a single deck (Deck 7, see Supplementary Materials), where designated healthcare areas were identified. All remaining areas were occupied by crew staff. On Deck 7, only the cabins along the perimeter sides (right side and left side) of the ship were designated for COVID positive patients, and a single pathway was identified to enable passage from one side of the ship to the other (unsafe zone). Cabins located at the center were allocated for support activities and not for patient healthcare. Four of these, referred to as “back-up” cabins, were allocated for patients who tested negative for SARS-CoV-2, cured and pending discharge. This solution enabled the optimization of bed space turnover times insofar as, once vacant, patient cabins could be sanitized, ready for use for new admissions. Initially, a first module was defined and used (module 1) for hospitalization, consisting of 27 cabins and a total of 31 beds (13 on the left side and 14 on the right side), including four two-beds for patients with particular needs (e.g., same family unit, etc.). This was followed by a second module (module 2) consisting of 25 cabins (12 on the left side and 13 on the right side), all single bed, adjacent to the previous one. 

The “unsafe” zone was suitably delimited, and included part of the ship’s bow, patient cabins, the fresh air section, the lifts, facilities for the sanitization of trolleys, the undressing area, and rooms for the storage of waste and bed linen that presented a risk of infection. Safe areas were positioned on either end of the unsafe areas of both modules, consisting of the following areas: staff dressing area, operational center, healthcare direction, clean bed linen room, infirmary, pharmacy, small kitchen hospital, oxygen tank storage, healthcare material, and personal protective equipment (PPE) storage. Both modules were divided in the same way. Pathways and rooms were marked with clear signage to favor orientation on the ship and prevent conditions of risk infection. The “fresh air area” and the “catering area” were used by patients three times a day after meals, so that they could socialize. These areas were also used by nursing staff for information and educational activities on social distancing, hand hygiene, and correct mask use. Sessions were also run in English for foreign patients.

Specialized healthcare staff allocated to work on the ship hospital guaranteed continuous healthcare (seven days a week, 24 h a day), and for each module, the team consisted of six doctors, ten nurses, and five healthcare assistants. Both modules were served by a single scientific director/clinical coordinator, a healthcare director, a healthcare assistant coordinator, a nursing coordinator, a coordinator for healthcare professionals, and five healthcare assistants. The scientific director/clinical coordinator served as the medical director of the facility, and was tasked with patient triage and the preparation and updating of the pre-hospitalization assessment sheets on the ship hospital, port of Genoa. They were also tasked with providing patient clinic services, on call 24 h for clinical emergencies and/or second opinions. Healthcare staff sourced from different local hospital facilities had a variety of skills and fields of expertise, thus guaranteeing high-level patient care. 

### 2.5. The Preparation and Serving of Meals

On board, staff prepared meals for patients directly on the ship on Deck 6, in accordance with a defined procedure, subject to dietary approval by the hospital healthcare direction. Particular attention was placed on the religion and culture of patients; diets were also personalized according to culinary traditions (e.g., rice instead of pasta), in addition to clinical and religious factors. Foods were prepared and packaged in single portion trays, which were placed in thermal food containers. Staff fitted with all necessary PPE transported them by trolley to Deck 7, using the service lift. Upon arrival at the floor, only the containers were removed. Single trays were then assembled and distributed to single cabins. Patients ate their meals inside the cabin. Food waste was collected directly by patients, in a suitably sealed bag. At the end of each meal, an operator would collect them in a biobox fitted onto a trolley, requesting each patient to deposit their own bag inside it. After the service, both the containers and trolleys were cleaned and sanitized using nebulized chlorine solutions.

## 3. Infection Risk Management Procedures

### 3.1. Hygiene Behavioral Measures

Various hygiene behavioral measures were implemented in the hospital ship area and external areas, using standard/universal and specific precautions for the ship hospital. Anyone leaving the hospital premises and entering other areas of the ship was required to perform all decontamination actions required before accessing “safe” areas. Whenever healthcare staff came into contact with patients, they were required to wear an FFP3 mask (N99/KN99), visor, and high protection suit; they also received periodic training. The ship’s crew also underwent periodic training on the infective risk of COVID-19. All infection risk control measures were developed on board, in collaboration with the reference hospital infection committee nurse; posters were created to encourage compliance with hygiene behavioral regulations. Hospital ship premises were cleaned and disinfected at least twice a day by designated and suitably trained staff equipped with appropriate protective equipment, by implementing a specific sanitization protocol in accordance with national healthcare directives.

### 3.2. Management of Waste and Bed Linen Presenting a High Infection Risk

All waste generated in the Deck 7 area was considered hazardous medical waste presenting a risk of infection, including all waste which, prior to the pandemic situation, would have been considered urban waste and subjected to separate waste collection. Before being removed from the ward, containers from the unsafe zone were decontaminated externally with a chlorine solution. These were then stored in a designated room located abaft the ward. The transport trolley was taken to Deck 3 (the garage) via the stern lift and then delivered to the designated depot. The pathway and trolleys were then suitably sanitized. Dirty bed linen from the isolation area was conferred in two bags: an inner biodegradable one (different from the one normally used for uninfected bed linen) and an outer red bag. Handling methods were the same as those for waste. Waste and dirty bed linen were removed from the ship four times a week by specialized firms.

### 3.3. Maintenance of the Hospital Ship

Ordinary maintenance of the areas and systems was guaranteed by on-board staff who received training regarding all infection risk prevention procedures. All maintenance operations were carried out in the absence of patients whenever possible; suitable PPE was always used.

### 3.4. Epidemiological Surveillance

This activity was carried out by a healthcare operator who collected and elaborated reports on a daily basis on hospitalized patients, containing data on contagion, symptoms, comorbidities, etc. An overall report on patients was prepared and submitted on a daily basis, containing the numbers of admissions, discharges, home discharges, patients discharged for admission to other facilities, and re-admissions.

### 3.5. Description of Admitted Patients

From 23 March to 18 June 2020, 191 patients were admitted onto the ship, with a male majority (57.07%), from different hospital facilities in Liguria and mostly from the Genoese metropolitan area. The average age was 56 years, ranging in total from 15 to 87 years. The average length of hospitalization was 15 ± 8.9 days (range 2–57 days, median 13 days). Some admitted patients had pre-existent comorbidities, most frequently diabetes, hypertension, or chronic obstructive pulmonary disease (COPD). During their stay at the hospital, only 4.71% of patients requested psychological support. No cases of voluntary discharge were recorded, or any episodes of self-harm or violence towards operators. Table 2 contains the main characteristics of patients.

Five patients who had been previously admitted onto the ship had to be transferred to the hospital after a number of days, ranging from a minimum of 9 up to 57 days later (Table 3). Two returned to the hospital ship in less than a week from their previous transfer.

## 4. Conclusions

The ship was fitted out to become a hub at a metropolitan level and then, at a regional level, a facility where COVID-19 positive patients requiring low to medium intensity care could be hospitalized. This lightened the care burden of hospitals, freeing up facilities and healthcare staff for patients in the acute phase of the illness. One hundred and ninety-one patients were treated over three months, with a favorable outcome in all cases, confirmed by their recovery and negative swab results. Patients were provided with high-level healthcare, guaranteed by the multi-disciplinary nature of the clinical competencies available. Patients also benefited from a multitude of actions (such as the possibility to access protected fresh air and catering areas), contributing towards psychological and physical well-being for more rapid functional recovery. Moreover, evidence-based design studies demonstrate that the presence of spaces for physical and psychological well-being is strategic for all users, and it also positively influences the work performance of the healthcare staff [15].

The results obtained also come from the effective collaboration with regional hospitals, and with the coordination of patients by telephone through the above-mentioned triage system. Hospitals dispatched non-routine medication and sent all documentation of clinical-diagnostic exams carried out during patients’ hospitalization. To the best of our knowledge, this is the only example in the world in which a passenger ship was transformed into a hospital ship for COVID patients in such a short space of time and with such excellent clinical results. This transformation has involved various professional figures, with technical or clinical skills, underlying the importance of a multidisciplinary approach, which also characterized hospitals’ reorganization during the COVID-19 pandemic [16]. A ship fitted out in this way is also ready to be used by other geographical areas. All patients who transited on the ship tested negative, and no cases of contagion between healthcare workers or crew members were recorded.

## Figures and Tables

**Table 1:** Ship hospital pre-hospitalization assessment sheet.

Pre-Hospitalization Assessment Sheet for the Ship Hospital, Port of Genoa			
Name			
Surname			
Date and place of birth			
Hospital			
Ward			
YES	NO	Patient with current COVID-19 infection diagnosis	
		Colonized patient/patient infected with multidrug resistant organisms (MDRO)	
		Colonized patient/infected with *Clostridium difficile* (CD)	
		Dementia	
		Drug dependence	
		Psychiatric disorder	
		Claustrophobia	
		Oxygen therapy Litres/min:	
		Has the patient’s clinical and diagnostic assessment been completed?	
		Are the patient’s clinical conditions stable?	
		Does further hospitalization require low intensity care?	
		Is the patient able to walk autonomously and are they self-sufficient in daily functions?	
		Is the patient awake, oriented, and cooperative?	
		Is hospital pharmacological therapy and clinical monitoring still required?	
		Has a treatment cycle been prepared for the patient for the presumed duration of the disease?	
		Has necessary clinical documentation been prepared for the patient, including a detailed treatment program?	
		Does healthcare documentation contain the phone numbers of relatives or family members authorized by the patient?	
First positive swab date:		Second swab date (if any):	Third swab date (if any):
Specify any comorbidities and/or essential anamnestic data: Signature and stamp of the Discharging Physician Physician’s Mobile Phone:			

**Table 2:** Characteristics of patients hospitalized on the COVID hospital ship.

Characteristics of Patients Hospitalized	% (*n*)
**Symptoms Upon Admission**	
Cough	7.85 (15)
Fever	2.62 (5)
Dyspnea	3.66 (7)
**Level of Self-Sufficiency**	
Autonomous	99.48 (190)
Oriented	99.48 (190)
Ambulatory	100 (191)
Ambulatory with aid	6.81 (13)
**Comorbidities**	
At least three	3.14 (6)
From three to five	6.28 (12)
More than five	4.71 (9)
Visual impairments	3.14 (6)
Hearing impairments	1.05 (2)
**Brass Index**	
0–10 Low risk	98.43 (188)
11–19 Medium risk	1.57 (3)
≥20 high risk	0.00 (0)
*Clostridium difficile* infection	2.09 (4)

**Table 3:** Characteristics of patients who were transferred to the hospital.

Patient	Age	Gender	Reason of Re-Admission to Hospital	Days Admitted in Hospital Ship Before Transfer to Hospital
Patient 1	58	Female	Diverticulosis (returned to ship after seven days)	20
Patient 2	63	Male	Acute pancreatitis	57
Patient 3	82	Male	Transferred to hospital for diagnostic investigation	37
Patient 4	59	Male	Taken to ED due to chest pains (returned to ship after two days)	10
Patient 5	75	Female	Fell, probable fracture of right leg, knee and femur	9

## Supplementary Materials

The following are available online at https://www.mdpi.com/1660-4601/17/23/8976/s1, Figure S1: Plan of Deck 7.
