# Supplementary material for: Transformation of a Ferry Ship into a Ship Hospital for COVID-19 Patients
Source: Int J Environ Res Public Health. 2020 Dec 2;17(23):8976. doi: 10.3390/ijerph17238976 (PMC7730349; doi:10.3390/ijerph17238976)
Supplement: Supplementary file 1 [file ijerph-17-08976-s001.pdf]

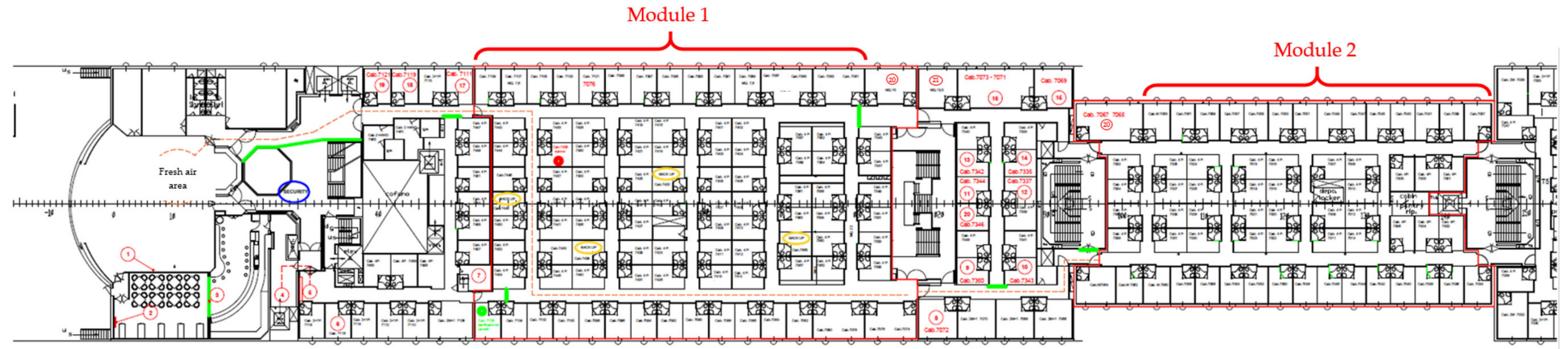

Legend:

1 - Patients buffet
2 - TV lounge
3 - Bulkhead isolating TV room
4 - Patients Meal Delivery Area
5 - Patient Meal and Tray Composition Area
Bulkhead (green line)

6 - Hospital kitchen
7 - Deposit & Storage " Ideal Service " Products
8 - Clean Linen
9 & 10 - Locker Room " Ideal Service ".
11 & 12 - Medical facilities
13 & 14 - Pharmacy

15 - Health Director Office
16 - Operative Centre
17 - Dirty Linen
18 & 19 - Special Wastes
20 – Undressing area
21 – Dressing area
